# Synthetic data generation with Worley–Perlin diffusion for robust subarachnoid hemorrhage detection in imbalanced CT Datasets

**DOI:** 10.1007/s11548-025-03482-2

**Published:** 2025-09-02

**Authors:** Zhongyang Lu, Tao Hu, Masahiro Oda, Yutaro Fuse, Ryuta Saito, Masahiro Jinzaki, Kensaku Mori

**Affiliations:** 1 Graduate School of Informatics, Nagoya University, Furo-cho, Chikusa-ku, Nagoya, Aichi Japan; 2 Information Technology Center, Nagoya University, Furo-cho, Chikusa-ku, Nagoya, Aichi Japan; 3 Department of Neurosurgery, Graduate School of Medicine, Nagoya University, 65 Tsurumai-cho, Showa-ku, Nagoya, Aichi Japan; 4 Department of Radiology, Keio University School of Medicine, 35 Shinanomachi, Shinjuku-ku, Tokyo, Japan; 5 Research Center for Medical Bigdata, National Institute of Informatics, 2-1-2 Hitotsubashi, Chiyoda-ku, Tokyo, Japan

**Keywords:** Imbalanced classification, Worley–Perlin noise, Diffusion model, Subarachnoid hemorrhage

## Abstract

****Purpose**:**

In this paper, we propose a novel generative model to produce high-quality SAH samples, enhancing SAH CT detection performance in imbalanced datasets. Previous methods, such as cost-sensitive learning and previous diffusion models, suffer from overfitting or noise-induced distortion, limiting their effectiveness. Accurate SAH sample generation is crucial for better detection.

****Methods**:**

We propose the Worley–Perlin Diffusion Model (WPDM), leveraging Worley–Perlin noise to synthesize diverse, high-quality SAH images. WPDM addresses limitations of Gaussian noise (homogeneity) and Simplex noise (distortion), enhancing robustness for generating SAH images. Additionally, $\hbox {WPDM}_{\text {Fast}}$ optimizes generation speed without compromising quality.

****Results**:**

WPDM effectively improved classification accuracy in datasets with varying imbalance ratios. Notably, a classifier trained with WPDM-generated samples achieved an F1-score of 0.857 on a 1:36 imbalance ratio, surpassing the state of the art by 2.3 percentage points.

****Conclusion**:**

WPDM overcomes the limitations of Gaussian and Simplex noise-based models, generating high-quality, realistic SAH images. It significantly enhances classification performance in imbalanced settings, providing a robust solution for SAH CT detection.

## Introduction

**Fig. 1:**
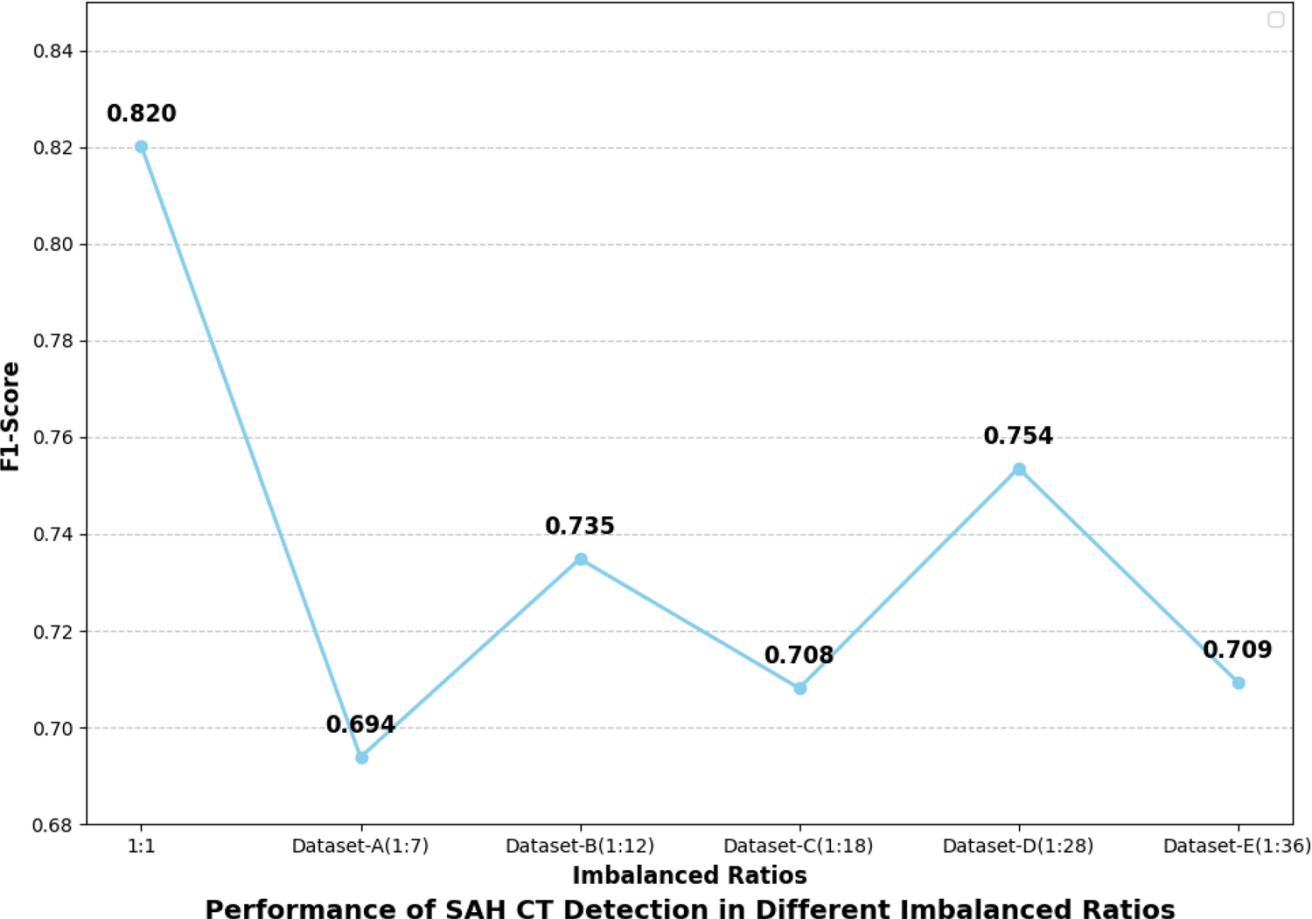
Evaluation results of DenseNet-121 trained with cross-entropy loss on original datasets (without generated samples) with varying imbalance ratios. The horizontal axis represents the degree of imbalance in the training set (as detailed in Table 1), while the vertical axis shows the F1-score on a consistent test set

Subarachnoid hemorrhage (SAH) is a severe form of hemorrhagic stroke with a significantly high risk of sudden mortality [1]. Early detection and proactive treatment are essential to reduce SAH-related mortality [2]. In patients with suspected SAH, noncontrast CT is the first diagnostic tool due to its high sensitivity [1, 2]. However, as the time from the onset to hospital admission increases, the sensitivity of CT scans gradually decreases [3], increasing the likelihood of misdiagnosis. Furthermore, the growing reliance on CT diagnostics has significantly increased the volume and complexity of cases, substantially increasing the workload of radiologists. This underscores the urgent need for a rapid and highly accurate automated SAH CT detection system to ease the burden of radiologists and provide critical support to emergency physicians.

Although there are some studies on SAH CT detection, they largely overlook the issue of data imbalance and are based heavily on costly segmentation masks [4]. At the same time, deep learning performance decreases significantly under data imbalances or insufficient samples (Fig. 1). Unfortunately, data imbalance is prevalent in medical scenarios, for instance, in detecting SAH CT, where collecting disease-specific cases is much more expensive than nondisease cases. This imbalance often prevents classifiers from adequately learning pathological features, biasing models toward negative classifications and increasing the risk of misdiagnosis with potentially irreversible consequences.

To address data imbalanced challenges, Cui et al. [5] introduced a novel weighting scheme based on the number of effective samples for each class. DRO-LT [6] applies robust optimization to estimate worst-case class distributions, expanding decision boundaries for minority classes through a push-pull strategy. LDAM-DRW [7] utilizes generalization error theory, mathematically demonstrating that increasing decision boundaries for minority classes reduces the generalization error. It proposes boundary adjustment rules tied to sample sizes for each class. ImbSAM [8] introduced a class-based optimization algorithm that takes advantage of previous knowledge to improve generalization for tail classes, demonstrating simplicity and efficacy. Although successful in natural image tasks, these methods often struggle with the unique challenges of medical imaging. The high cost of obtaining SAH data further limits sample availability, leading to representation learning issues from insufficient data. Lu et al. [9] proposed a DenseNet-121- and LSTM-based framework with CB loss [5] to address the same issue with us. While LSTM-based frameworks have demonstrated reliable performance on balanced datasets, their robustness tends to degrade significantly under severe class imbalance, a scenario frequently encountered in real-world clinical applications. Singh et al. [10] systematically explored the effectiveness of integrating LSTM architectures with various imbalance mitigation techniques within the context of multiple resident activity recognition. Singh’s findings [10] and those of Lu [9] suggest that although these strategies can partially alleviate the negative effects of imbalance, they often fail to fully resolve performance degradation caused by substantial data scarcity and skewed class distributions.

**Fig. 2:**
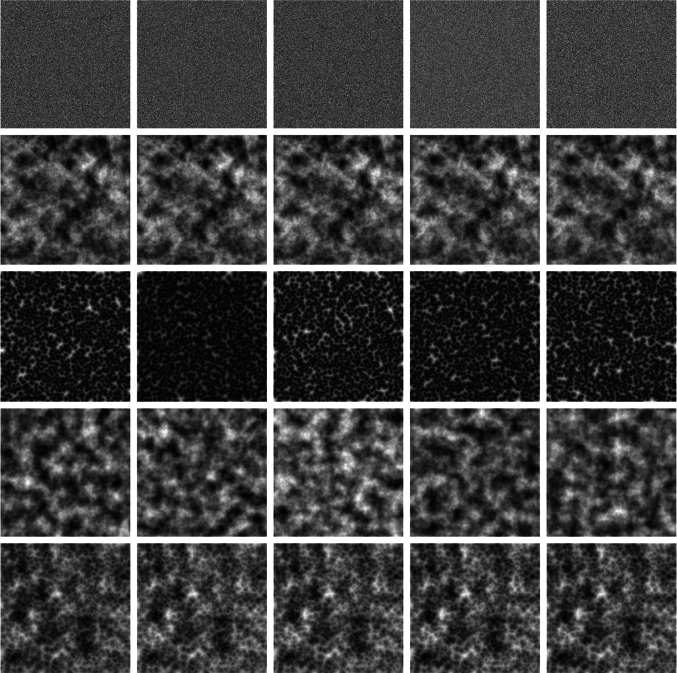
Various noise types. From top to bottom: Gaussian noise, Simplex noise, Worley noise, Perlin noise, and the proposed Worley–Perlin noise (ours)

Generative models, particularly the denoising diffusion probabilistic models (DDPMs) [11, 12], offer an effective solution to address sample scarcity. Wu et al. [13] proposed MedSegDiffV2, a novel Transformer-based diffusion framework for medical image segmentation. Wyatt et al. [12] proposed AnoDDPM for brain tumor segmentation, revealing that Gaussian noise in diffusion models uniformly perturbs images, minimally affecting low-frequency components and limiting output diversity. Our experiments also found that Simplex noise [12] can introduce excessive uneven perturbations, causing distortions in the generation of SAH images. In Fig. 2, we compare Gaussian noise, Simplex noise, and our proposed Worley–Perlin noise. Gaussian noise is white noise with a flat spectrum, meaning its power is evenly distributed across frequencies. This characteristic allows diffusion models to explore a broader potential distribution during the early stages of the process. However, during subsequent denoising steps, the noise is gradually guided toward high-probability regions, which leads to convergence and limits diversity. In contrast, Simplex noise, which is nonuniform, improves the convergence problem and hence, as demonstrated in Fig. 11, disrupts the structural integrity of the generated brain images.

**Fig. 3:**
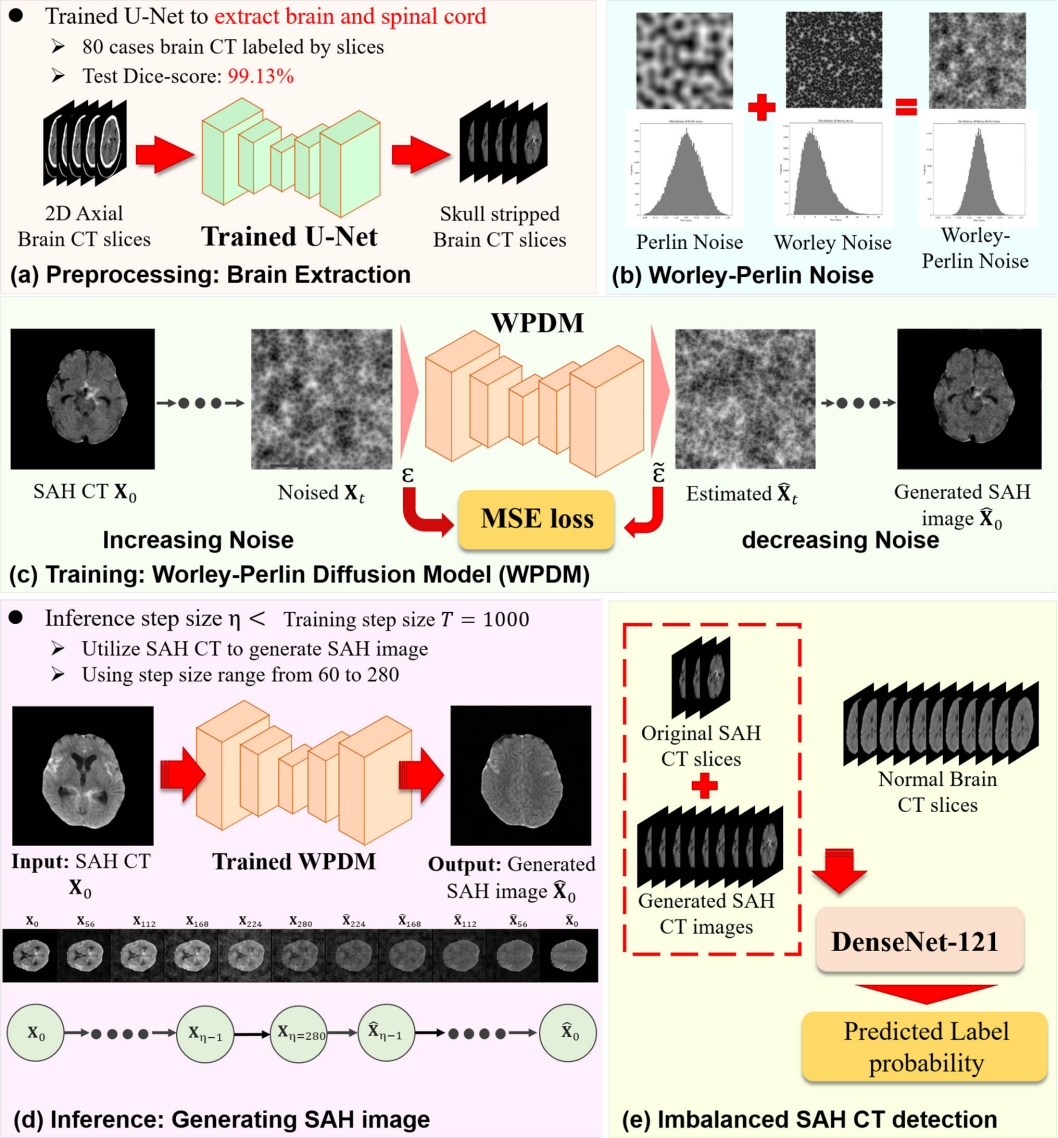
Flowchart of the proposed WPDM for imbalanced SAH CT detection: (**a**) Brain and spinal cord extraction using trained U-Net model with a Dice score of 0.991. (**b**) Combination of Worley and Perlin noise to create a novel noise. (**c**) WPDM uses MSE loss to iteratively denoise and reconstruct synthetic SAH CT slices. (**d**) Trained WPDM generates realistic SAH images for data augmentation. (**e**) DenseNet-121 is trained on a combined dataset of original and synthetic CT images to address data imbalance and predict label probabilities

**Fig. 4:**
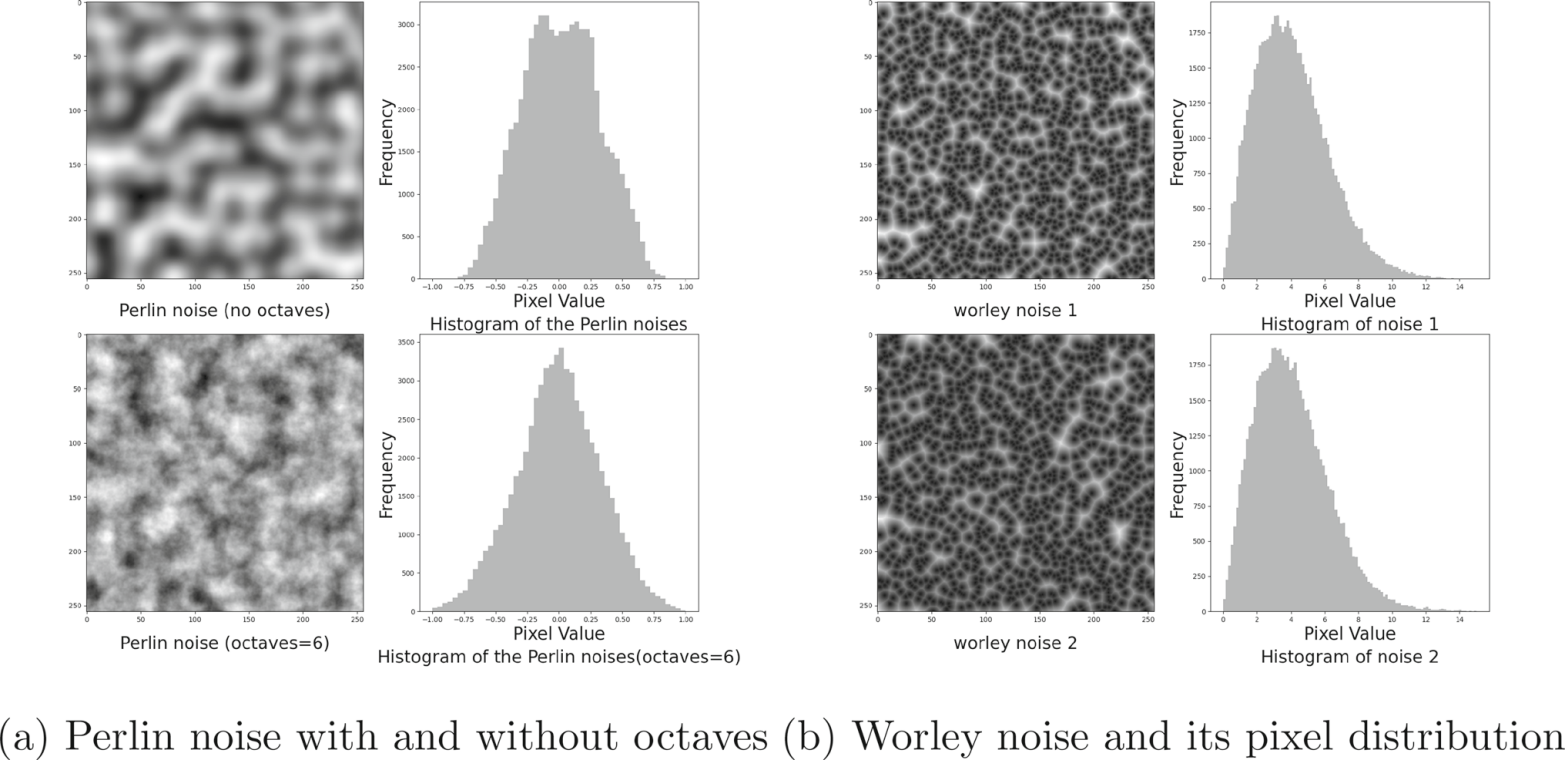
Perlin noise and Worley noise. (**a**) Octaves represent the number of layers of perturbation. It can be observed that Perlin noise with multiple octaves exhibits finer details due to the accumulation of perturbations. (**b**) The pixel distribution of Worley noise exhibits a skewed characteristic

This study introduces the Worley–Perlin Diffusion Model (WPDM) designed to generate high-quality SAH CT images, as illustrated in Fig. 3. Worley–Perlin noise is a fine-grained, nonuniform distribution that reduces convergence risks while preserving sample morphology and the original data distribution. WPDM **generates diverse SAH samples** to enhance training datasets, enabling **robust and accurate SAH CT detection**, even under extreme data imbalance. The key contributions of this work include:

- *High-quality image generation:* WPDM synthesizes complex-textured SAH CT slices, addressing limitations of Gaussian and Simplex noise to improve robustness and diversity.
- *Addressing data imbalance:* WPDM significantly improves classification performance on imbalanced datasets by generating diverse samples, enhancing generalization and detection.
- *State-of-the-art performance:* WPDM outperformed ImbSAM by 2.3 F1-score points on a 1:36 imbalance dataset, demonstrating its effectiveness for imbalanced classification tasks.

**Fig. 5:**
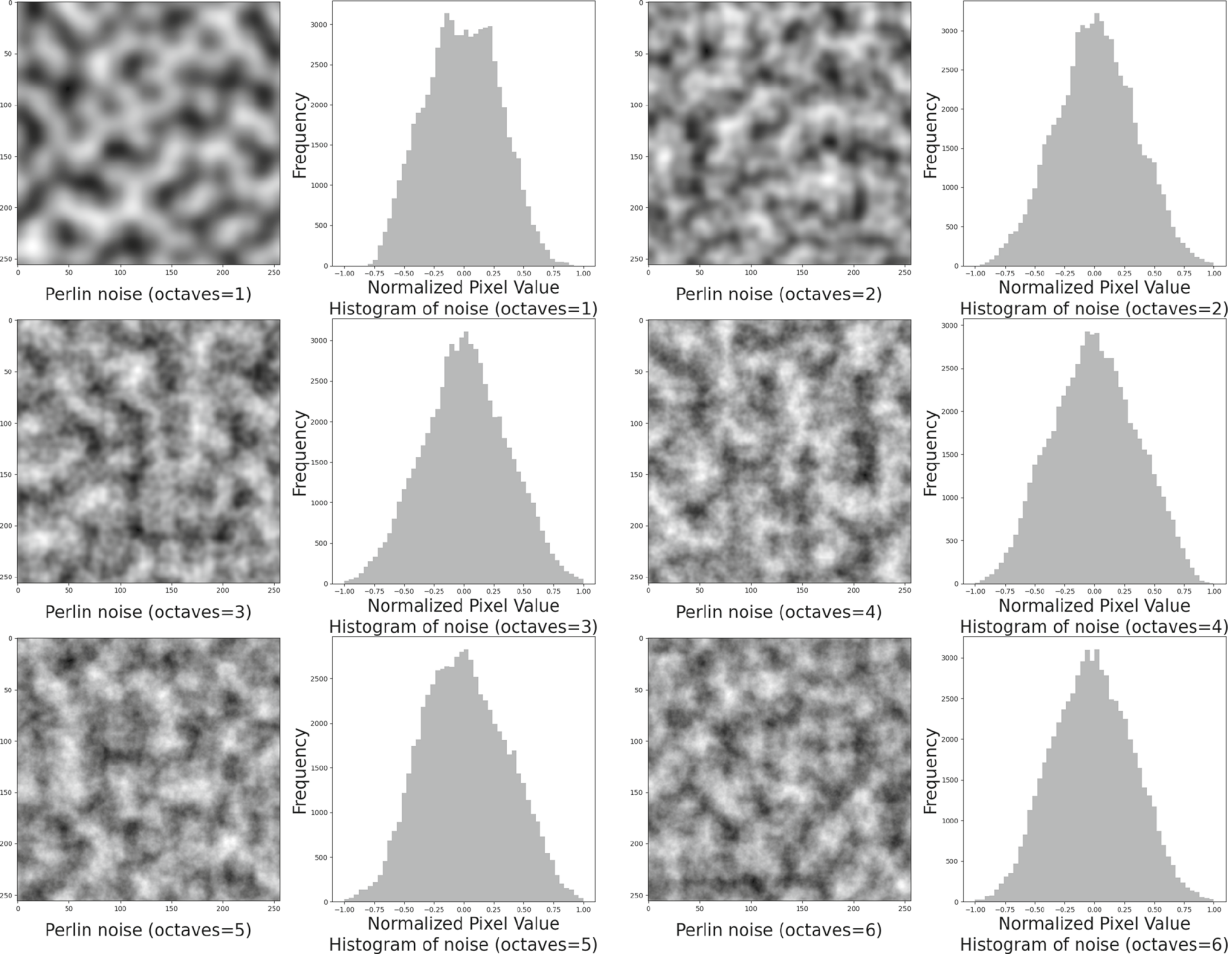
Perlin noise with different numbers of octaves. Octaves here refer to halving the noise granularity and overlaying it onto the original noise. As the number of octaves increases, higher frequency components are introduced, resulting in finer details and more small-scale textures

## Methods

### Brain extraction

To eliminate interference from soft tissues and cranial bones, we trained a common U-Net model in an additional brain CT dataset with fully annotated 2D axial slice to segment brain tissue and connected regions of the cervical spinal before performing generation and classification tasks. Then, we employ it to conduct brain extraction on whole datasets. Before entering the data into the U-Net model, we standardized the pixel size to $512\times 512$ and normalized the pixel intensity values to a range of 0 to 1. As shown in Fig. 3(a), after processing with the trained U-Net segmentation model, we obtained brain CT images with the skull and irrelevant soft tissues removed, which were subsequently used for downstream tasks.

### Theoretical Basis of DDPM

The combination of diffusion models and deep learning can be traced back to Sohl-Dickstein et al. [14], who introduced a novel unsupervised learning approach inspired by nonequilibrium thermodynamic processes in physical systems. This model learns to generate complex data distributions from simple noise distributions by progressively adding and denoising noise.

Building on this foundation, DDPM integrates the principles of diffusion processes and variational inference, which employs a Markov chain to iteratively sample Gaussian noise. The model consists of two key components: a forward diffusion process and a reverse diffusion process.

*Forward diffusion process:* During training, a Markov chain progressively adds noise to the data, transforming the original data distribution into a pure Gaussian noise distribution. The forward diffusion process is mathematically represented as:

1 $$\begin{aligned} q(\textbf{x}_t \mid \textbf{x}_{t-1}) := \mathcal {N}(\textbf{x}_t; \sqrt{1-\beta _t} \textbf{x}_{t-1}, \beta _t \textbf{I}), \end{aligned}$$

which highlights the core steps of the diffusion model, the sampling operation $\textbf{x}_t \sim \mathcal {N}(\sqrt{1-\beta _t}\textbf{x}_{t-1}, \beta _t\textbf{I})$, which explicitly demonstrates that the random variable $\textbf{x}_t$ is a result of this distribution. $q(\textbf{x}_{t} \mid \textbf{x}_{t-1})$ means that the image $\textbf{x}_{t}$ in the time step *t*, after noise addition, depends only on the state $\textbf{x}_{t-1}$ from the previous time step. The image $\textbf{x}_{t}$ follows a Gaussian distribution with mean of $\sqrt{1-\beta _t} \textbf{x}_{t-1}$ and covariance of $\beta _{t}\textbf{I}$. $\beta _{t}$ and $\textbf{I}$ means variance of the noise added at time step *t* and identity matrix, respectively.

*Reverse diffusion process:* The model performs denoising via a reverse Markov chain to recover data samples from Gaussian noise. Each sampling step depends on the distribution of the current step. The backward diffusion process is described by:

2 $$\begin{aligned} p_{\mathbf {\theta }}(\textbf{x}_{t-1} \mid \textbf{x}_t) := \mathcal {N}(\textbf{x}_{t-1}; \mu _{\theta }(\textbf{x}_t, t), \Sigma _{\theta }(\textbf{x}_t, t)), \end{aligned}$$

which can be considered as the reverse process of Eq. 1, Similarly, $\textbf{x}_{t-1}$ is generated from the previous distribution with a mean of $\mu _{\theta }(\textbf{x}_t, t)$ and a variance of $\Sigma _{\theta }(\textbf{x}_t, t)$. $\Sigma _{\theta }(\textbf{x}_t, t)$ is the Gaussian distribution covariance matrix, which is also a function of $\textbf{x}_t$ and *t*, and parameterized by $\mathbf {\theta }$.

### Worley–Perlin noise

Worley noise is commonly used to create patterns such as stone textures, droplet distributions, and cellular structures [15]. Perlin noise is a gradient noise technique that is used to generate continuous terrains in video games [16]. As shown in Figs. 4 and 5, single-frequency Perlin noise appears to be too coarse and lacks sufficient detail. In contrast, the layered Perlin noise effectively mitigates this issue. However, Worley noise is overly sparse and has limited effectiveness in perturbing the image. Simply layering Worley noise not only results in significantly increased computational cost, but also yields limited improvements.

**Fig. 6:**
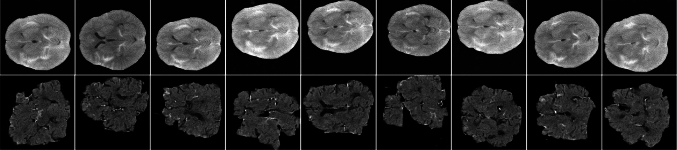
Generation of SAH CT images from pure noise. The top row shows homogeneous samples generated using DDPM with Gaussian noise. The bottom row displays mode collapse and generation failures from AnoDDPM with Simplex noise

Gaussian noise is commonly used in DDPMs; however, it is not the only viable option [12, 17]. Choosing the appropriate noise type is crucial for optimizing performance in the target task. As discussed in Sect.  1 and in Fig. 2, previous noise models have shown limitations, prompting us to propose Worley–Perlin noise, a nonuniform but fine-grained noise. Its non-uniformity promotes diversity in generated samples, while fine granularity preserves the original structure, preventing distortion. This approach leverages the strengths of Gaussian and Simplex noise while addressing their respective shortcomings. Additionally, we introduce a novel synthesis method that integrates Worley and Perlin noise, followed by zero-centering and normalization to ensure compatibility with the diffusion process. This transformation is mathematically defined as follows:

3 $$\begin{aligned}&\textbf{x}_{p} \sim \mathcal {P},\quad \textbf{x}_w \sim \mathcal {W}, \quad \textbf{x}_m \sim \mathcal {M}, \quad \textbf{x}_m \nonumber \\&\quad = \lambda \sum _{i=0}^{i=6} \Phi (\textbf{x}_{p}^{i}) + \gamma \Phi (\textbf{x}_{w}). \end{aligned}$$

Perlin noise and Worley noise are derived from interpolation and geometric computations, making it difficult to explicitly define their probability density functions (PDFs) [15, 16]. Instead, we implicitly represent the probability distributions of Perlin noise value $\textbf{x}_p$ and Worley noise value $\textbf{x}_w$ as $\mathcal {P}$ and $\mathcal {W}$, respectively. The probability distribution of the proposed Worley–Perlin noise value $\textbf{x}_m$ is denoted as $\mathcal {M}$. As shown in Fig. 5, accumulating Perlin noise produces finer-grained and more detailed noise. By stacking it six times, we achieve the desired effect while avoiding excessive computational overhead. The $\Phi (\cdot )$ is zero-centering and normalization. $\lambda$, $\gamma$ are hyperparameters that adjust the weights of the Perlin and Worley noise components, respectively.

### Worley–Perlin diffusion model

We replaced Gaussian noise with Worley–Perlin noise while maintaining the DDPM framework for both diffusion and sampling processes. To validate WPDM’s effectiveness in generating SAH images, we trained it on 2D axial SAH CT slices, learning the characteristic distribution of SAH CT images. The WPDM is defined as $\boldsymbol{\epsilon }_\theta (\textbf{x}_t, t)$, which predicts noise at the time step *t*. Its optimization follows the same approach as DDPM. Detailed training and sampling processes are described in Algorithms 1 and 2.

During generation, the small sample size of SAH CT data often leads to homogeneous or failed samples when using Gaussian or Simplex noise (Fig. 6). To address this, we introduced a perturbation-based generation approach with a step size smaller than the maximum training step. That described in Algorithm 3 enables the generation of SAH samples to balance the positive and negative sample ratio in the data set for downstream tasks.

**Algorithm 1:**
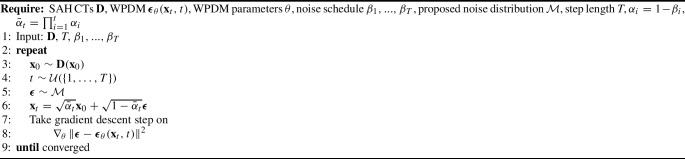
WPDM Training

**Fig. 7:**
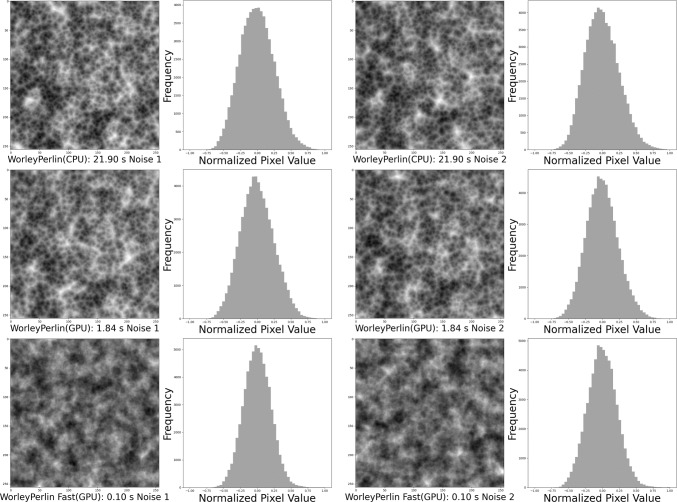
Worley–Perlin noise fast, our optimized version. Compared to the unoptimized version, it achieves nearly 18 times faster generation, significantly enhancing the model’s efficiency

**Algorithm 2:**
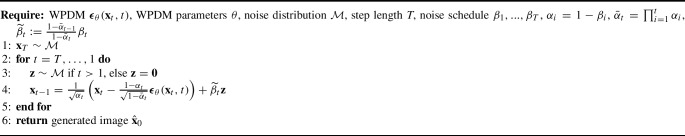
WPDM Sampling

**Algorithm 3:**
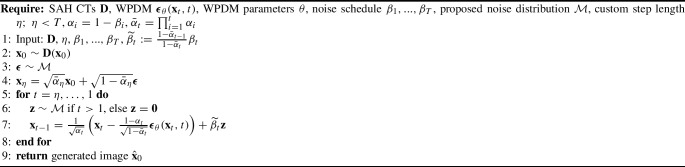
WPDM-based SAH Generation

### A faster variant of Worley–Perlin diffusion model

We optimize the Perlin noise generation process by focusing on 2D computations. Specifically, we directly computed gradients and interpolations on a 2D grid, reducing unnecessary complexity and computational overhead. Using PyTorch, we leveraged efficient tensor operations and GPU acceleration, making the method suitable for deep learning workflows. This optimization significantly improved the generation efficiency while maintaining the quality of the generated samples and the accuracy of downstream classification tasks. In Fig. 7, we visualize the optimized version of Worley–Perlin Noise, referred to as Worley–Perlin noise Fast, alongside the original Worley–Perlin Noise, based on both CPU and GPU computations. We also compare the generation time for every two samples. The titles of the noise images in Fig. 7 indicate that Worley–Perlin noise Fast reduces generation time from 3.67 s to 0.02 s per two images. Both versions were generated in the same device environment. We named the WPDM using Worley–Perlin Noise Fast as $\hbox {WPDM}_{\text {Fast}}$.

### Imbalanced SAH CT detection

As illustrated in Fig. 3(e), for the task of detecting 2D axial SAH CT slices under imbalanced data conditions, we utilized datasets containing 25 SAH cases along with normal head CT images at varying imbalance ratios. Furthermore, we used trained WPDM (using step sizes ranging from 60 to 280) to generate synthetic SAH samples, balancing the dataset so that the number of SAH and normal samples became nearly equal. The rebalanced datasets were subsequently used to train a DenseNet-121 model, resulting in a robust and high-performance detector for SAH CT slices.

**Table 1:** Details of training datasets used in imbalanced classification

Training datasets	SAH cases	Normal cases	SAH slices	No-SAH slices	pixels	Approximate imbalanced ratio
#Dataset-A	25	50	436	2,860	$512 \times 512$	1:7
#Dataset-B	25	100	436	5,572	$512 \times 512$	1:12
#Dataset-C	25	150	436	7,986	$512 \times 512$	1:18
#Dataset-D	25	250	436	12,574	$512 \times 512$	1:28
#Dataset-E	25	325	436	15,882	$512 \times 512$	1:36

## Experiments and results

### Experimental settings

#### Dataset

The datasets used in this study, provided by the National Institute of Informatics, Japan, include head CT images from 33 SAH patients and a large number of normal CT scans. All images were annotated by experienced physicians to indicate the presence or absence of SAH and are in DICOM format with a pixel size of $512 \times 512$.

*Brain extraction task.* We divided 80 fully annotated 2D axial slices into three subsets: 55 cases for training, 10 for validation, and 15 for testing. The brain and connected spinal cord regions of each slice were manually annotated as the truth of the ground.

*SAH images generation task.* SAH images generation task. We trained the generation model using 436 SAH-positive CT slices from 25 SAH cases. During generation, we produced additional SAH samples to rebalance each dataset based on its imbalance ratio.

*SAH CT detection in imbalanced datasets.* As shown in Table 1, we constructed training datasets with varying imbalance ratios by mixing 25 SAH cases (436 positive and 494 negative slices) with different numbers of normal cases: 50 normal cases for a 1:7 ratio, 100 normal cases for a 1:12 ratio, 150 normal cases for a 1:18 ratio, 250 normal cases for a 1:28 ratio, 325 normal cases for a 1:36 ratio.

These datasets evaluated the method’s effectiveness under various imbalance conditions. The validation set included 3 SAH cases (57 positive and 55 negative slices) and 6 normal cases (274 slices). The test set comprised 5 SAH cases (109 positive and 69 negative slices) and 10 normal cases (522 slices).

**Table 2:** Hyperparameters of WPDM

Diffusion steps	1000
Resized image size	$256\times 256$
Epoches	3000
Batch size	16
Number of attention head	2
Learning rate	1e-5
Noise schedule	linear
Optimizer	AdamW
Hyperparameters $\lambda ,\gamma$	0.5, 1

**Table 3:** Hyperparameters of classifier

Resized image size	$256\times 256$
Backbone	DenseNet-121
Epoches	500
Loss function	Cross-entropy
Batch size	128
Learning rate	1e-2
Optimizer	SGD
Weight decay	1e-4
Momentum	0.9

**Table 4:** Performance comparison across different methods in Imbalanced ratio 1:7 (Dataset-A)

**Method**	**Accuracy (%)**	**AUC**	**Precision**	**Recall**	**F1-score**
Original data (DenseNet-121 + CE)	$91.43 \pm 1.29$	$0.918 \pm 0.023$	$0.787 \pm 0.092$	$0.637 \pm 0.042$	$0.699 \pm 0.022$
CB Loss (CVPR 2019 [5])	$92.57 \pm 0.74$	$0.924 \pm 0.015$	$0.834\pm 0.029$	$0.655 \pm 0.069$	$0.732\pm 0.039$
LDAM-DRW (NeurIPS 2019 [7])	$93.34 \pm 0.63$	$\underline{0.945 \pm 0.011}$	$0.844 \pm 0.063$	$0.710 \pm 0.055$	$0.768 \pm 0.022$
CB_DenseNet_LSTM (SPIE MI 2021 [9])	$90.75 \pm 0.55$	$0.934 \pm 0.013$	$\mathbf {0.917 \pm 0.048}$	$0.629 \pm 0.044$	$0.744 \pm 0.021$
DRO-LT (ICCV 2021 [6])	$91.94 \pm 1.25$	$0.925 \pm 0.033$	$0.795 \pm 0.080$	$0.664 \pm 0.083$	$0.718 \pm 0.048$
ImbSAM (ICCV 2023 [8])	$93.89 \pm 0.96$	$\mathbf {0.952 \pm 0.022}$	$0.837 \pm 0.080$	$\underline{0.767 \pm 0.038}$	$\underline{0.797 \pm 0.020}$
DDPM (NeurIPS 2020 [11])	$92.91 \pm 2.08$	$0.935 \pm 0.009$	$0.804 \pm 0.103$	$0.739 \pm 0.022$	$0.767 \pm 0.050$
AnoDDPM (CVPR 2022 workshop [12])	$91.51 \pm 2.64$	$0.935 \pm 0.019$	$0.736 \pm 0.148$	$\mathbf {0.778 \pm 0.076}$	$0.745 \pm 0.054$
WPDM (ours)	94.23 ± 0.73	$0.944 \pm 0.011$	$\underline{0.894 \pm 0.036}$	$0.716 \pm 0.036$	$0.794 \pm 0.027$
$\hbox {WPDM}_{\text {Fast}}$(ours)	$\mathbf {94.29 \pm 0.14}$	$0.944 \pm 0.009$	$0.867 \pm 0.044$	$0.752 \pm 0.047$	$\mathbf {0.804 \pm 0.008}$

The results presented in the table are the mean and standard deviation calculated from five repeated experiments. CE means cross-entropy loss. The bold indicates the best result, and the second-best is underlined

#### Implementation details

Our main experiment consists of two parts: (1) **Training Generative Model.** We trained WPDM using 436 SAH-positive 2D CT slices from 25 SAH cases to learn the distribution of SAH CT images; (2) **SAH CT Detection in rebalanced datasets.** All experiments included simple data augmentation through affine transformations during training and were conducted on 2D axial CT slices. Detailed experimental parameters are summarized in Tables 2 and 3. Experiments were conducted on the PyTorch platform with CUDA 11.6, using a single NVIDIA Quadro RTX 8000 GPU with 48GB memory. Performance was evaluated using standard metrics including recall, precision, F1-score, and AUC.

**Fig. 8:**
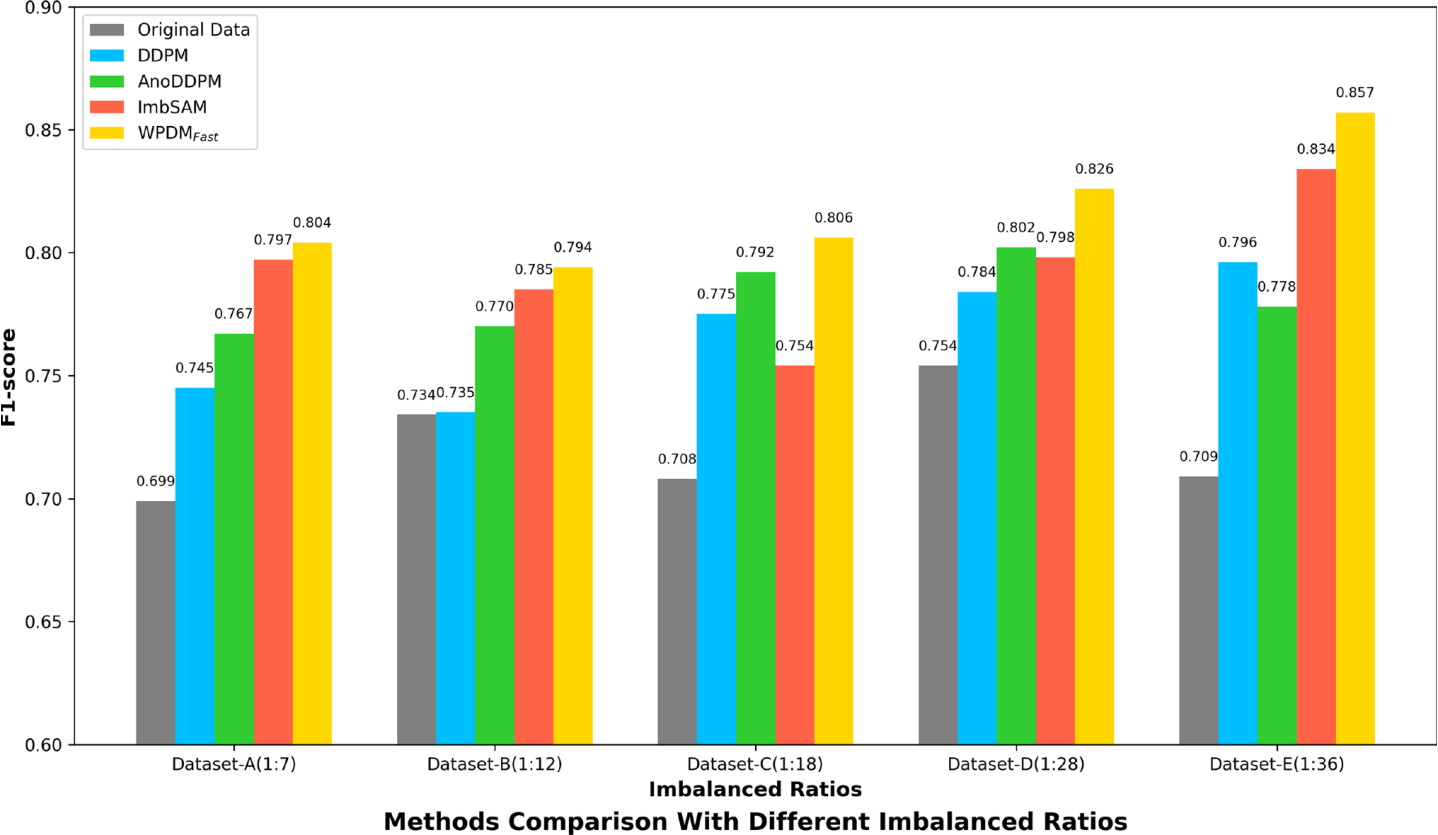
Comparison of F1-scores for different methods across datasets with varying imbalance ratios. The x-axis represents the original imbalance ratios in their training datasets. $\hbox {WPDM}_{\text {Fast}}$ consistently outperforms other methods, achieving the highest F1-scores across all imbalance scenarios

### Experimental results

#### Qualitative evaluation

We conducted experiments on various imbalanced datasets to evaluate the effectiveness of our WPDM and its variant $\hbox {WPDM}_{\text {Fast}}$ in improving SAH CT detection under imbalanced data conditions. The results are summarized in Table 4 and Fig. 8.

For imbalanced classification tasks, the F1-score is a more reliable metric than accuracy. $\hbox {WPDM}_{\text {Fast}}$ achieved an F1-score of, slightly outperforming WPDM, demonstrating superior performance in balancing precision and recall. WPDM and $\hbox {WPDM}_{\text {Fast}}$ outperformed most previous methods, such as LDAM-DRW and DRO-LT, with $\hbox {WPDM}_{\text {Fast}}$ exceeding the state-of-the-art ImbSAM.

**Fig. 9:**
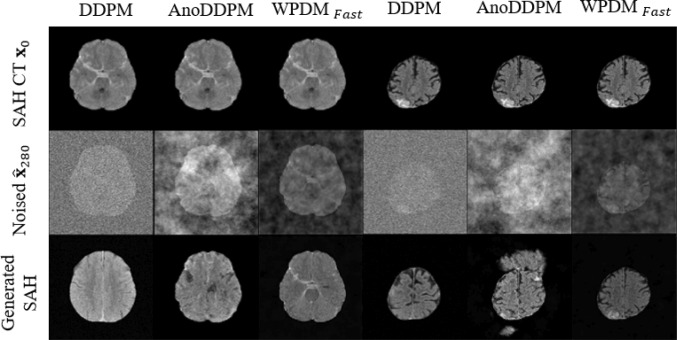
Comparison of generative model results. Two representative SAH CT images were selected to compare the outputs of DDPM (**Gaussian noise**), AnoDDPM (**Simplex noise**), and $\hbox {WPDM}_{\text {Fast}}$ (**Worley-Perlin noise Fast**)

**Table 5:** Comparison in Fréchet inception distance of generative models

	DDPM [11]	AnoDDPM [12]	WPDM(ours)	$\hbox {WPDM}_{\text {Fast}}$(ours)
**FID Score** $\downarrow$	24.625	26.137	**19**.**350**	19.817

The bold indicates the best result, and the second-best is underlined

As shown in Fig. 1, data imbalance generally degrades performance, but different methods exhibit varying robustness under different imbalance ratios. Some methods exhibit greater robustness in handling more severe imbalance scenarios. To validate our method’s effectiveness across various imbalance ratios, we compared $\hbox {WPDM}_{\text {Fast}}$ with representative methods from previous works, including generative approaches like DDPM and AnoDDPM, and the state-of-the-art imbalanced classification method ImbSAM. Using $\hbox {WPDM}_{\text {Fast}}$ as the representative model, we compared their F1-scores to evaluate its superiority in the target task. The results in Fig. 8 show that $\hbox {WPDM}_{\text {Fast}}$ outperformed all previous methods at all imbalance ratios, achieving an especially notable F1-score of 0.857 at the extreme ratio of 1:36.

**Fig. 10:**
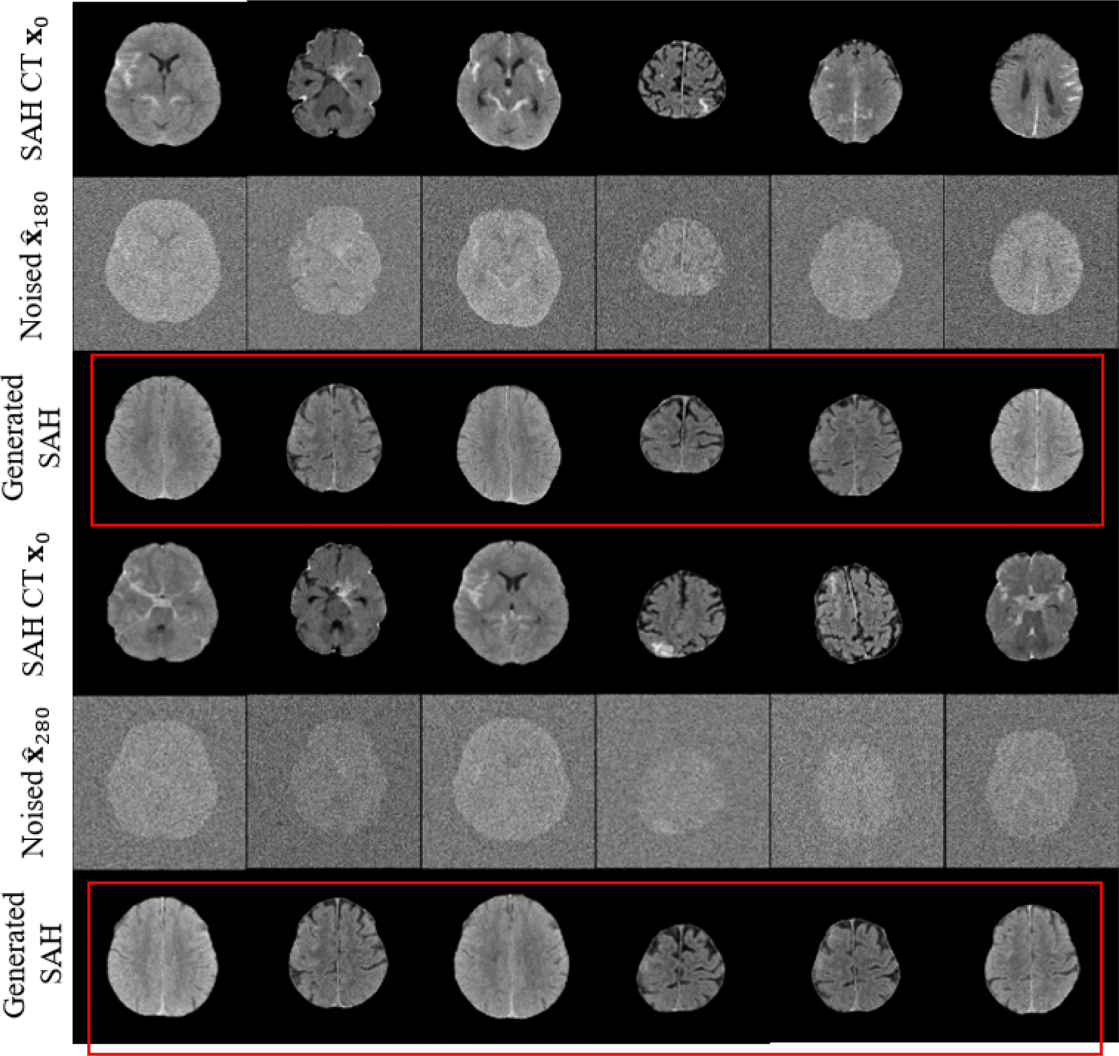
Visualization of generated SAH images across different noise levels using DDPM (**Gaussian noise**). Rows correspond to original images, added noise, and generated results. As shown within red boxs, the generated images exhibited convergence

#### Quantitative evaluation

**Fig. 11:**
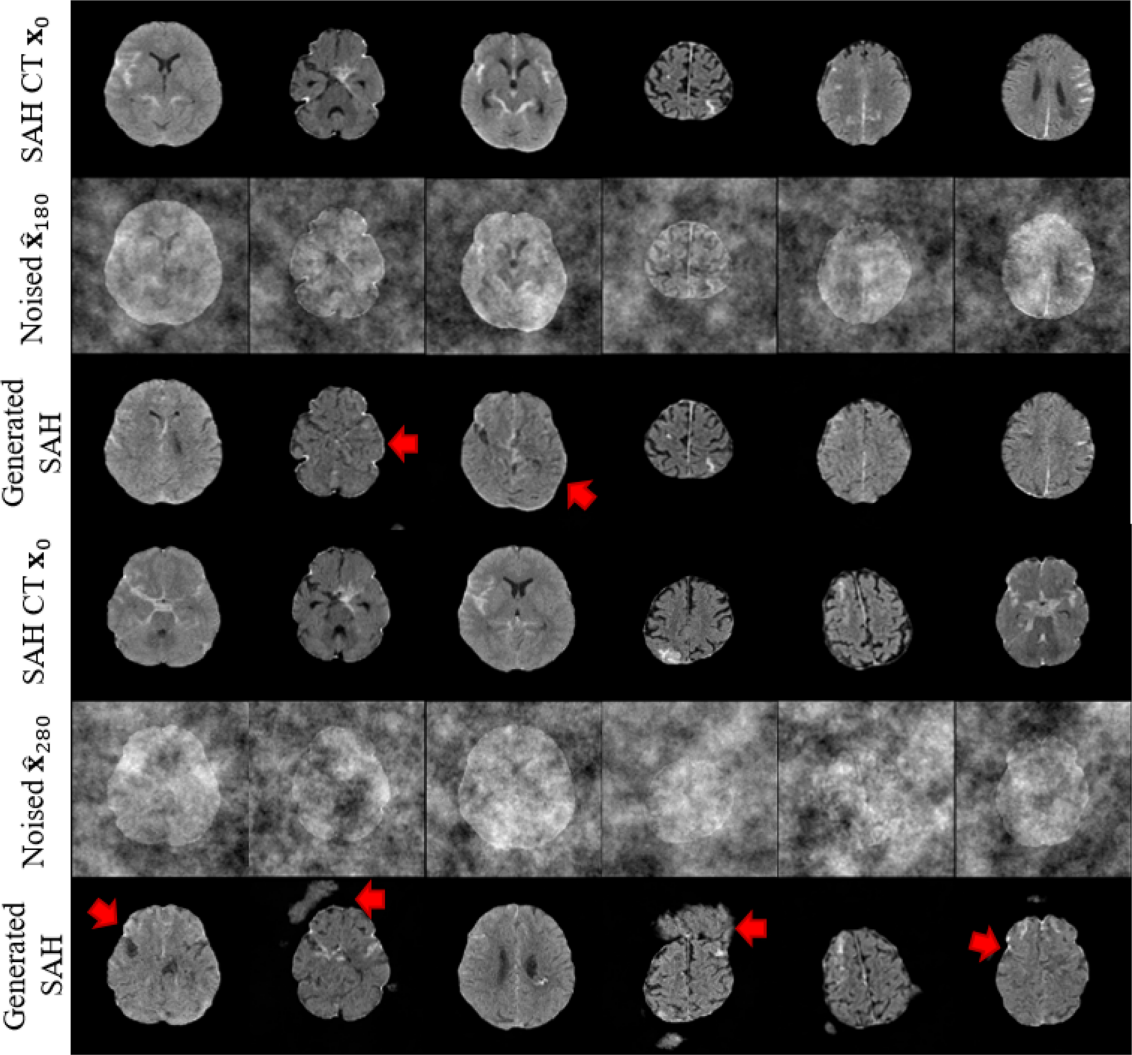
Visualization of generated SAH images across different noise levels using AnoDDPM (**Simplex noise**). Rows correspond to original images, added noise, and generated results. As indicated by the red arrow, some generated images displayed damaged brain structures, with SAH features being insufficiently pronounced

**Fig. 12:**
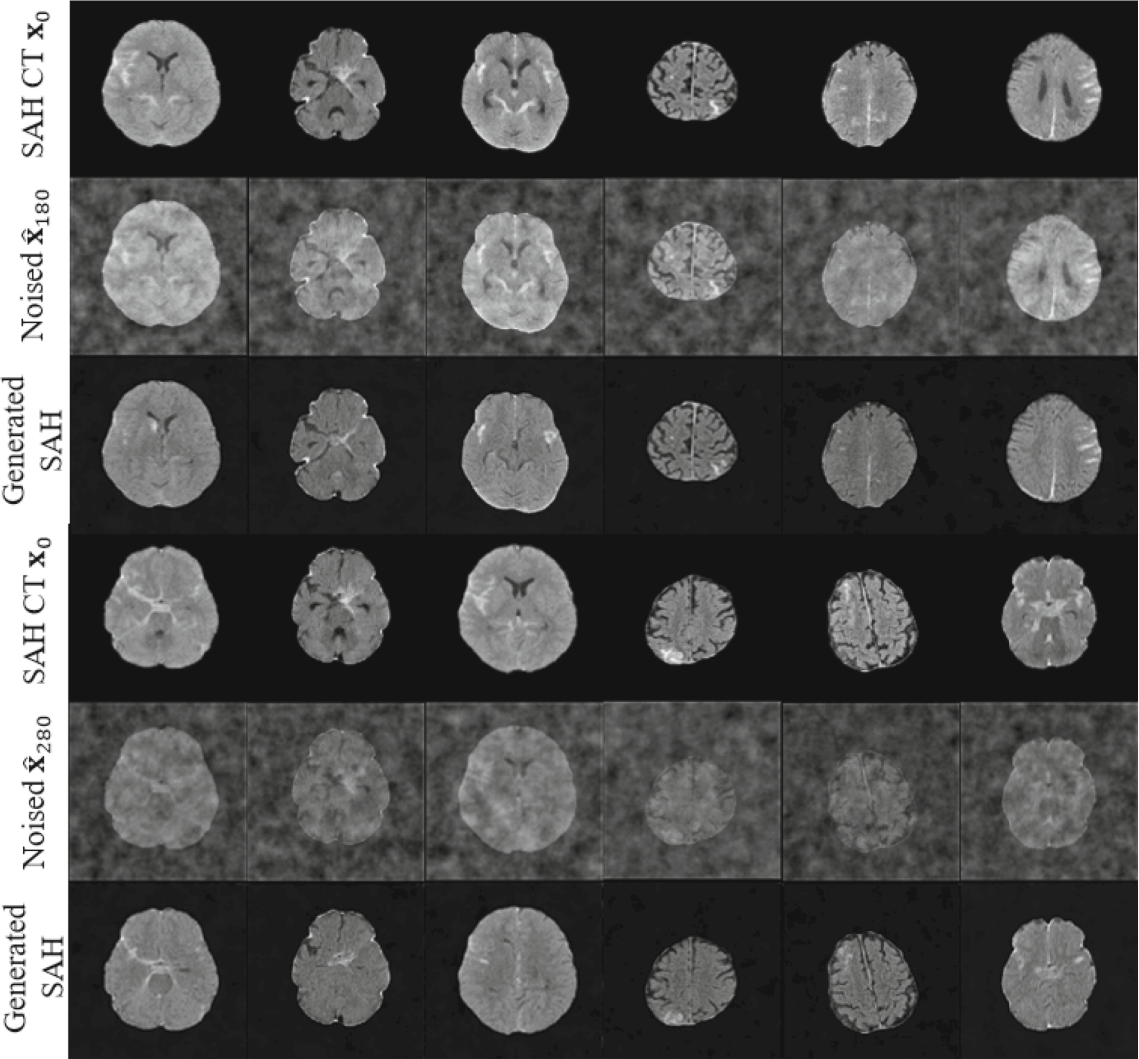
Visualization of generated SAH images across different noise levels using $\hbox {WPDM}_{\text {Fast}}$ (**Worley–Perlin noise Fast**). Rows correspond to original images, added noise, and generated results. Rows correspond to original images, added noise, and generated results, demonstrating the model’s ability to denoise while preserving structural details

In Figs. 10, 11, and 12, we visualize the SAH images generated by the generative models DDPM [11], AnoDDPM [12], and $\hbox {WPDM}_{\text {Fast}}$ (with the results of WPDM being nearly equivalent). To highlight the strengths and weaknesses of each method, we selected two prediction step sizes, $\eta =180$ and $\eta =280$. In Fig. 9, we provide a comparative summary of the generated results from these methods. Additionally, to better quantify the quality of the generated images, we evaluated the generative performance using the Fréchet Inception Distance (FID). Unlike traditional FID models used for natural image evaluation, we adopted Wang’s model, which is trained on the RSNA intracranial hemorrhage detection dataset, as the evaluation model [18]. The results, shown in Table 5, indicate that our proposed method achieved the best performance.

## Discussion

As shown in Table 4, our proposed WPDMs outperformed cost-sensitive learning approaches such as CB Loss [5] and DRO-LT [6]. While LDAM-DRW [7] improves robustness by minimizing the gap between empirical and generalization errors, its performance is limited by the small training dataset of 436 SAH CT slices.

Generative methods increase data diversity through synthetic samples, but sample quality is critical. DDPM [11] produces overly homogeneous samples (Fig. 10), and AnoDDPM [12] suffers from mode collapse, generating samples with unclear SAH features or structural failures (Fig. 11). In extreme imbalance scenarios, poor-quality synthetic samples can degrade performance (Fig. 8). WPDMs address these challenges by generating diverse and effective samples, even with limited training data (Fig. 12). These samples significantly improve classifier performance, as evidenced by both quantitative (Table 5) and qualitative evaluations (Table 4). WPDMs improve classifier performance across varying imbalance ratios by adding high-quality synthetic samples to training sets.

ImbSAM [8] improves performance by incrementally training on majority and minority samples, smoothing the optimization surface. However, due to the scarcity of SAH CT samples, ImbSAM struggles to outperform WPDM, especially in extreme imbalance scenarios Fig. 8). WPDMs generate structurally similar but distinct samples, introducing novel SAH features not present in original datasets. This reduces learning difficulty and significantly enhances detector robustness and performance.

Furthermore, internal comparisons indicate that $\hbox {WPDM}_{\text {Fast}}$ achieves a balanced recall and precision, though its image generation quality (FID) is slightly lower than WPDM. However, the overall performance difference is within the margin of error. The main advantage of $\hbox {WPDM}_{\text {Fast}}$ is its improved generation efficiency.

Despite its advantages, WPDMs have limitations. The generated samples lack detail resolution compared to Gaussian noise-based DDPM, and the synthetic SAH features are somewhat constrained, likely due to feature coupling during representation learning. Addressing these issues will be the focus of future work.

## Conclusion

This paper introduces WPDM and its accelerated version, $\hbox {WPDM}_{\text {Fast}}$. These models exhibit high robustness in generating SAH CT images, addressing limitations of Gaussian and Simplex noise. Experiments on imbalanced SAH CT detection demonstrate WPDM significantly improves classification performance. By generating realistic synthetic CT images with clear striated features, WPDM enhances detection accuracy and robustness, offering a reliable solution for disease diagnosis in imbalanced datasets and supporting clinical research in small-sample scenarios, advancing precision medicine.
